# Pharmacokinetic evaluation of paclitaxel, albumin-binding paclitaxel, and liposomal-encapsulated albumin-binding paclitaxel upon gastric subserosal administration

**DOI:** 10.3389/fphar.2024.1381406

**Published:** 2024-06-06

**Authors:** Yoontaek Lee, Sun Mi Park, In Ho Song, Bom Sahn Kim, Hyun Soo Park, Byung Seok Moon, Hyung-Ho Kim

**Affiliations:** ^1^ Department of Surgery, Ewha Womans University Seoul Hospital, Ewha Womans University College of Medicine, Seoul, Republic of Korea; ^2^ Department of Nuclear Medicine, Ewha Womans University Seoul Hospital, Ewha Womans University College of Medicine, Seoul, Republic of Korea; ^3^ Department of Nuclear Medicine, Seoul National University Bundang Hospital, Seoul National University College of Medicine, Seongnam, Republic of Korea; ^4^ Department of Molecular Medicine and Biopharmaceutical Sciences, Graduate School of Convergence Science and Technology, Seoul National University, Seoul, Republic of Korea; ^5^ molim, Inc., Suwon, Republic of Korea; ^6^ Department of Surgery, Seoul National University Bundang Hospital, Seoul National University College of Medicine, Seongnam, Republic of Korea

**Keywords:** paclitaxel, liposome-encapsulated albumin-paclitaxel nanoparticles, gastric subserosal administration, nuclear medicine and molecular imaging, anticancer

## Abstract

**Introduction:** Systemic chemotherapy is typically administered following radical gastrectomy for advanced stage. To attenuate systemic side effects, we evaluated the effectiveness of regional chemotherapy using paclitaxel, albumin-paclitaxel, and liposome-encapsulated albumin-paclitaxel via subserosal injection in rat models employing nuclear medicine and molecular imaging technology.

**Method:** Nine Sprague Dawley rats were divided into three groups: paclitaxel (*n* = 3), albumin-paclitaxel nano-particles (APNs; *n* = 3), and liposome-encapsulated APNs (*n* = 3). [^123^I]Iodo-paclitaxel ([^123^I]I-paclitaxel) was synthesized by conventional electrophilic radioiodination using *tert*-butylstannyl substituted paclitaxel as the precursor. Albumin-[^123^I]iodo-paclitaxel nanoparticles ([^123^I]APNs) were prepared using a desolvation technique. Liposome-encapsulated APNs (L-[^123^I]APNs) were prepared by thin-film hydration using DSPE-PEG2000, HSPC, and cholesterol. The rats in each group were injected with each test drug into the subserosa of the stomach antrum. After predetermined times (30 min, 2, 4, 8 h, and 24 h), molecular images of nuclear medicine were acquired using single-photon emission computed tomography/computed tomography.

**Results:** Paclitaxel, APNs, and L-APNs showed a high cumulative distribution in the stomach, with L-APNs showing the largest area under the curve. Most drugs administered via the gastric subserosal route are distributed in the stomach and intestines, with a low uptake of less than 1% in other major organs. The time to reach the maximum concentration in the intestine for L-APNs, paclitaxel, and APNs was 6.67, 5.33, and 4.00 h, respectively.

**Conclusion:** These preliminary results imply that L-APNs have the potential to serve as a novel paclitaxel preparation method for the regional treatment of gastric cancer.

## Introduction

Gastric cancer one of the most prevalent malignancies in both Korea and worldwide, holding the fifth position in incidence globally and ranking as the fourth leading cause of death among all solid cancers (International Agency for Research on Cancer, 2022). In Korea, gastric cancer accounted for 10.8% of new cancer cases, ranking fourth with 26,662 reported cases, slightly trailing behind thyroid, lung, and colon cancers, as outlined in the report by the Korea Central Cancer Registry for the year 2020 (National Cancer Center, Korea Central Cancer Registry, 2022). Notably, owing to the national screening program, gastric cancer is often diagnosed at an early stage, termed early gastric cancer in Korea. Although radical gastrectomy with lymph node dissection is the standard treatment for gastric cancer, endoscopic submucosal dissection (ESD) is becoming increasingly common in patients with early gastric cancer (Guideline Committee of the Korean Gastric Cancer Association Development Working Group and Review Panel, 2019; Ono et al., 2021). However, despite the introduction of extended indications and the publication of long-term treatment results of ESD on early gastric cancer, recurrence and regional lymph node metastasis following ESD remain a challenge.

Adjuvant chemotherapy holds pivotal significance after radical gastrectomy in patients diagnosed with stage II or stage III gastric cancer (Sakuramoto et al., 2007; Sakamoto et al., 2009; Bang et al., 2012). Its primary aim is the eradication of residual cancer cells post-surgery, potentially diminishing the likelihood of recurrence and enhancing overall survival rates. Systemic chemotherapy, an established modality in gastric cancer treatment, employs pharmaceutical agents disseminated throughout the body to target and eradicate cancer cells, encompassing agents such as fluorouracil, oxaliplatin, capecitabine, paclitaxel, among others. However, traditional systemic chemotherapy via intravenous injection has the drawback of affecting both normal and cancerous cells, leading to undesirable side effects. In contrast, regional chemotherapy offers the enticing prospect of maintaining a high concentration of anticancer drugs in the tumor while minimizing side effects by confining the drug’s effects to specific regions or organs (Akamo et al., 1994; Kobayashi et al., 2014). This can be achieved through various methods, including intraperitoneal chemotherapy, transarterial chemoembolization, and intratumoral chemotherapy (Llovet et al., 2002; Kono et al., 2017).

**FIGURE 1:**
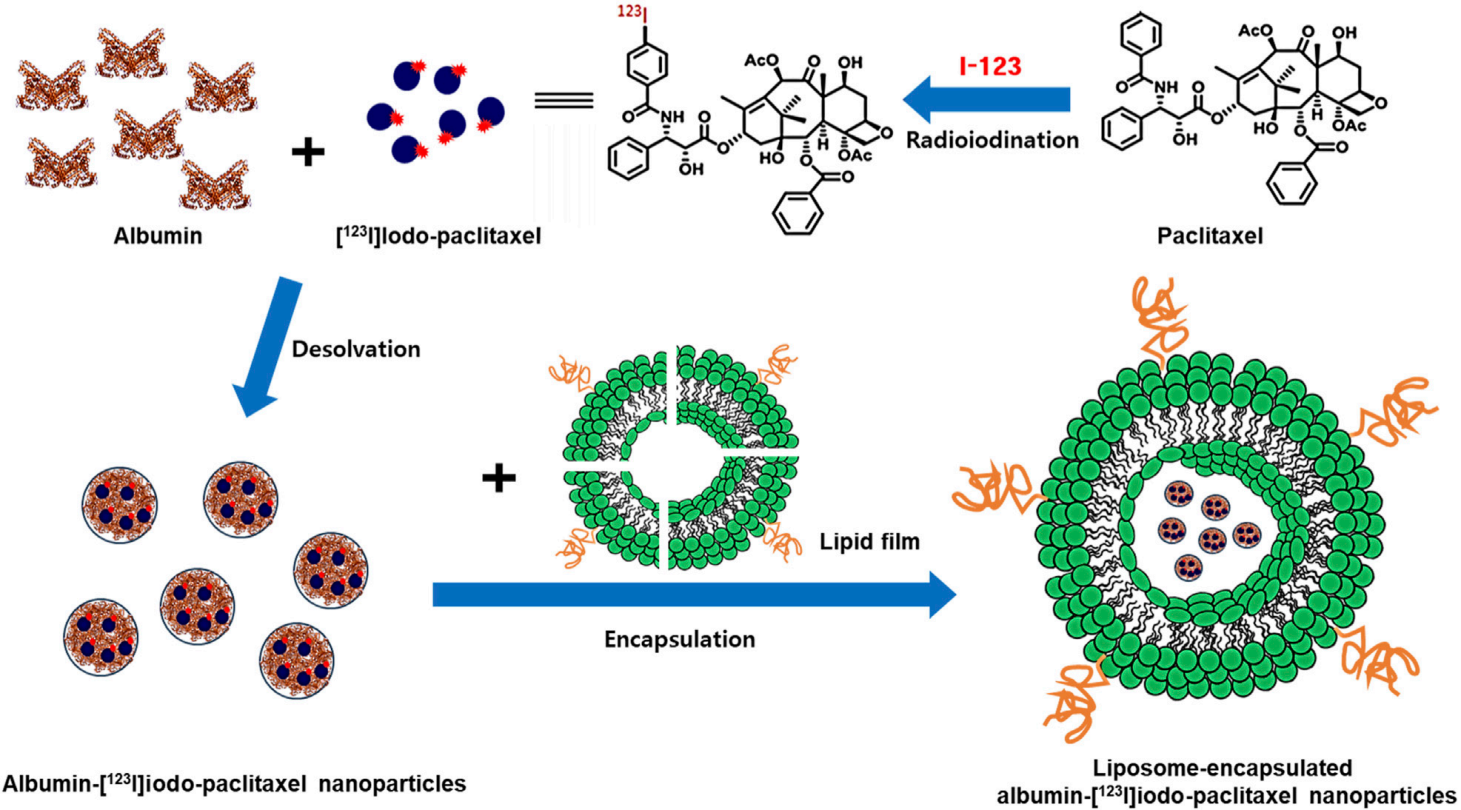
Schematics for the preparation of [^123^I]iodo-paclitaxel, albumin-[^123^I]iodo-paclitaxel nanoparticles and liposome-encapsulated albumin-[^123^I]iodo-paclitaxel nanoparticles.

This strategy is particularly promising for lipophilic anticancer drugs, which can help maintain high concentrations within the lymphatic system while maintaining low systemic blood concentrations (Worley et al., 2016). Furthermore, encapsulating anticancer drugs in liposomes allows for enhanced lymphatic concentration while preserving drug stability *in vivo* (Hua et al., 2010; Huang et al., 2018; Okamoto et al., 2019). The potential application of liposome-encapsulated albumin-paclitaxel in regional chemotherapy is promising due to its capacity to localize drug delivery at the tumor site while mitigating systemic side effects. Nonetheless, present research lacks investigations into regional chemotherapy for gastric cancer utilizing liposome-encapsulated albumin-paclitaxel, particularly regarding the dispersion of lipophilic anticancer drugs via subserosal injection. With this as the backdrop, we focused on evaluating and comparing the distribution of lipophilic anticancer drugs, including paclitaxel, albumin-paclitaxel, and liposome-encapsulated albumin-paclitaxel, within the body (Figure 1).

Furthermore, nuclear medicine and molecular imaging techniques utilizing radiolabeled drugs, such as positron emission tomography (PET) and single photon emission computed tomography (SPECT), offer distinct advantages over conventional approaches for assessing tissue pharmacokinetics (PK) (Vallabhajosula, 2007). This cutting-edge technology facilitates non-invasive, real-time visualization of biochemical processes at the cellular and molecular levels in living organisms. It enables the examination of the specific and selective binding of radiolabeled drug candidates to their targets, transcending the limitations of conventional PK studies. These methodologies allow for the visualization and quantification of tissue PK properties of drug candidates within specific organs and lesions of interest (Bergström et al., 2003; Lau et al., 2020). Image-based nuclear medicine and molecular imaging, consequently, emerges as a valuable tool for identifying nuanced biochemical or pathological events, facilitating disease diagnosis, predicting treatment outcomes, and contributing to effective treatment planning. Herein, nuclear medicine and molecular imaging were employed to investigate the effects of subserosal injections in rat models.

## Materials and methods

### General

Globulin and fatty acid-free human serum albumin were obtained from Sigma-Aldrich (#A3782, ≥99%, St. Louis, Missouri, USA). *tert*-Butylstannyl-paclitaxel (#FC-6080, SnBu_3_-paclitaxel, >95%) from Futurechem Co. (Seoul, Korea) was used as a precursor for radioiodination. Anhydrous ethanol was purchased from Merck (#1.00983, ≥99.9%, Rahway, New Jersey, USA) and ultrafiltration membranes were obtained from Sartorius (Goettingen, Germany). A PD-10 desalting column was purchased from GE Healthcare (CA, IL, USA). The iodine-123 labeled paclitaxel ([^123^I]I-paclitaxel) was synthesized by electrophilic iodination using a Na[^123^I]I solution with an oxidant. Albumin-[^123^I]iodo-paclitaxel nanoparticles were prepared using the desolvation technique and liposome-encapsulated albumin-[^123^I]iodo-paclitaxel nanoparticles were obtained using the thin-film method.

### Preparation of [^123^I]I-paclitaxel

**FIGURE 2:**
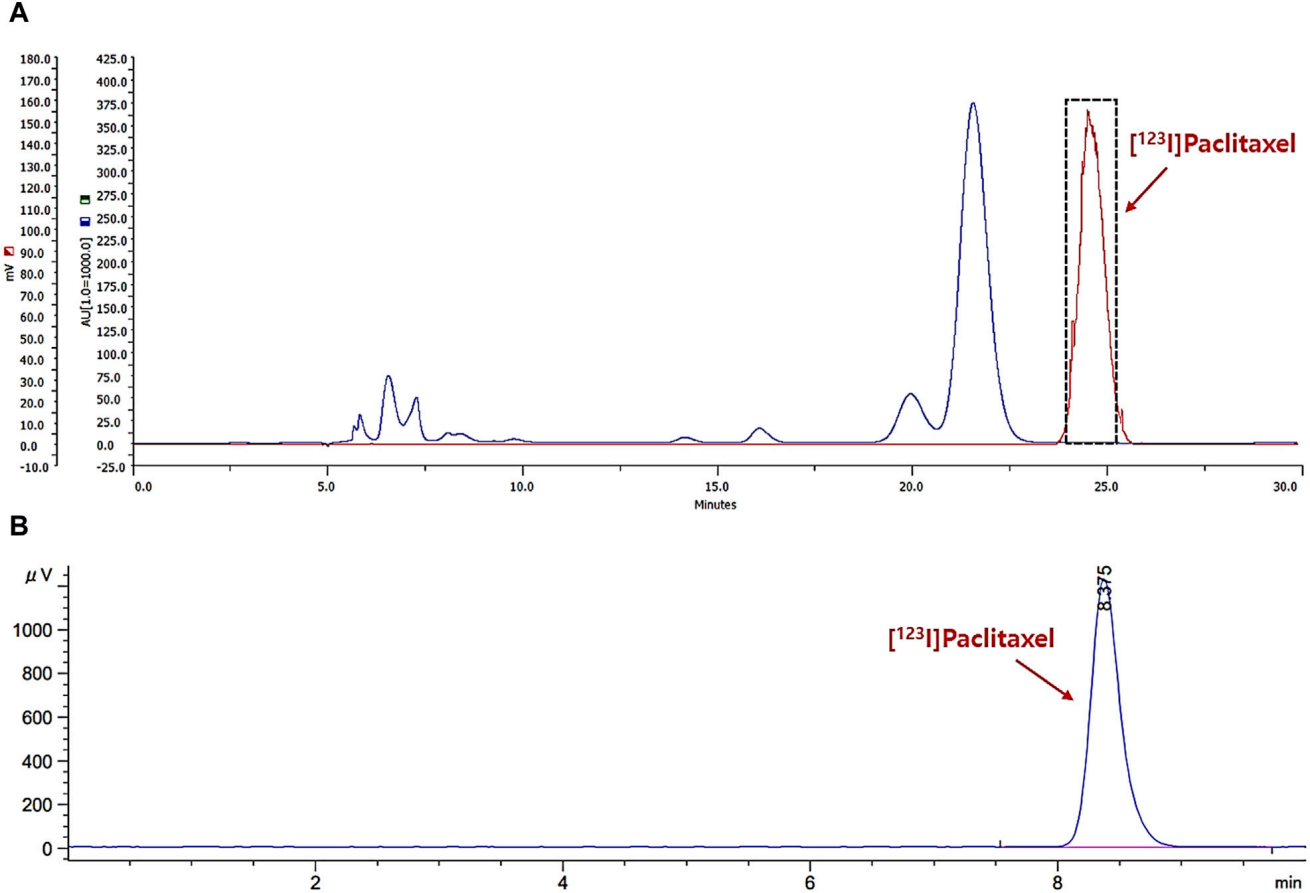
The chromatograms for separation profile of the reaction mixture **(A)**; gamma-ray: red, UV-230 nm: blue) and analysis profile of radiochemical purity **(B)**; gamma-ray).

[^123^I]I-paclitaxel was prepared by conventional electrophilic radioiodination using SnBu_3_-paclitaxel as a precursor, according to the literature, with a slight modification (Kiesewetter et al., 2003). Briefly, a solution of sodium hydroxide containing I-123 was added to a reaction vial containing chloramine-T trihydrate (0.1 mL, 1 mg/mL of water), 1 N HCl (50 μL), and SnBu_3_-paclitaxel (0.1 mg) in ethanol (0.1 mL). The mixture was then stirred at room temperature for 10 min. After dilution with 10 mL of water and 8.4% NaHCO_3_ solution (100 μL), the solution was loaded onto a C18 plus Sep-Pak cartridge, washed with water for injection (5 mL), and eluted with acetonitrile (1.5 mL). The eluted solution was diluted with 1.5 mL of water, filtered with a universal hydrophilic polytetrafluoroethylene (UHP) syringe filter (0.45 μm, 13 mm), and purified by semi-preparative high-performance liquid chromatography (HPLC; Gilson 322 system equipped with a UV-detector (230 nm) and NaI gamma-ray detector (LabLogic); XTerra RP18, 10 × 250 mm, 10 μm; 55% CH_3_CN/H_2_O, flow rate:3 mL/min). The collected solution, around 24.5 min of retention time (*t*_*R*_) from HPLC, was diluted with 17 mL of water, loaded onto a C18 plus Sep-Pak cartridge, and washed with water for injection (5 mL) to remove biologically unavailable HPLC solvent (Figure 2A). The desired product was then eluted with ethanol (1.0 mL). Finally, the ethanol solution was reduced to the desired volume using nitrogen streaming. The radiochemical purity of the final solution was confirmed by analytical HPLC (Figure 2B; Agilent 1260 system equipped with a UV-detector (230 nm) and NaI gamma-ray detector (Raytest); XTerra RP18, 4.6 × 250 mm, 5 μm; 60% CH_3_CN/H_2_O, flow rate: 1 mL/min).

### Preparation of albumin-[^123^I]iodo-paclitaxel nanoparticles ([^123^I]APNs)

Albumin-[^123^I]iodo-paclitaxel nanoparticles ([^123^I]APNs) were prepared using a previously described desolvation technique with slight modifications (Ruttala and Ko, 2015). In brief, fatty acid-free human serum albumin (1 mg/mL), practically devoid of globulin, was dissolved in distilled water and filtered through a UHP syringe filter (0.45 μm, 13 mm). The pH was adjusted to approximately nine using 1 M NaOH. Subsequently, dropwise addition of [^123^I]I-paclitaxel in ethanol solution (0.2 mL) was carried out into the albumin solution (2 mL) with stirring (500 rpm) at 3 min intervals for a total of 10 portions. The mixture was stirred for an additional 1.5 h. The mixture was separated by ultrafiltration using a 10-kDa ultrafiltration membrane or PD-10 desalting columns.

### Preparation of liposome-encapsulated APNs (L-[^123^I]APNs)

Liposome-encapsulated APNs (L-[^123^I]APNs) were prepared using the thin-film method described previously (Gill et al., 2012). In brief, L-[^123^I]APNs were encapsulated in liposomes by hydration of the dried lipid thin film. Hydro Soy phosphatidylcholine (HSPC), cholesterol, and DSPE-PEG2000 were dissolved in chloroform at a 90:10:5 M ratio. The organic solvent was evaporated under a stream of nitrogen gas to form a thin film at the bottom and wall of the vial. Freeze-drying was performed overnight to remove any traces of the remaining solvent. The dried film was hydrated with [^123^I]APNs solution and incubated at 42°C for 20 min. The multilamellar preparations obtained were resized by repeated extrusion through polycarbonate membrane filters. Free unentrapped [^123^I]APNs were removed by centrifugation at 5,000 rpm for 10 min. The supernatant was collected in a glass vial and stored at 4°C until further use. The sizes of [^123^I]APNs and L-[^123^I]APNs were determined by DLS using a Zetasizer Nano (*n* = 3, Malvern Instruments Ltd, United Kingdom). Drug entrapment efficiency (EE) was subsequently calculated using the following formula: EE = (radioactivity of drug in liposomes)/(radioactivity of feeding drug) × 100.

### Animals

Seven to nine-week-old male Sprague Dawley (SD) rats were obtained from Orient Bio Inc. (Seongnam, Korea) and maintained in a controlled environment. To allow them to adapt, the rats were acclimatized for 1 week, and maintained in air-conditioned quarters in a specific pathogen-free (SPF) environment during periods with free access to water and food. An SPF environment was maintained with approximately 55% humidity, 21°C temperature, 12 h light/dark cycle, *ad libitum* food and water, and housed in groups of two animals per cage. Prior to the experiments, we randomly divided ten mice into three groups with similar average body weight per group: paclitaxel (*n* = 3), albumin-paclitaxel nanoparticles (APNs, *n* = 3), and liposome-encapsulated APNs (L-APNs, *n* = 3). One rat was prepared as a contingency for unavoidable situations such as injection failure. Subserosal gastric injections were administered to rats under isoflurane anesthesia. Following an upper abdominal midline incision, each drug was injected into the subserosal antrum of the stomach. The muscular layers and skin incision were closed, and analgesics (ketoprofen, 5 mg/kg) were administered.

### SPECT/CT study

Each animal underwent whole-body SPECT/CT using a small animal-dedicated SPECT/CT system (NanoSPECT/CT; Mediso, Budapest, Hungary) with an axial field of view (FOV) of 10 cm and a transaxial FOV of 12 cm. The SPECT spatial resolution at the center of the FOV was 1.2 mm full-width at half maximum. Prior to the SPECT scan, CT was conducted with a semicircular full trajectory, maximum FOV, 723 projections, 55 kVp, 1000 ms, and 1:4 binning. For the biodistribution study, whole-body SPECT/CT images of the experimental mice were acquired at 30 min and 2, 4, 8, and 24 h (24–72 s per frame) after each injection of [^123^I]I-paclitaxel (15.7 ± 1.3 MBq), [^123^I]APNs (19.8 ± 2.6 MBq), and L-[^123^I]APNs (2.9 ± 0.1 MBq). During scanning, all animals were anesthetized with 2% isoflurane. The SPECT images were reconstructed using the iterative three-dimensional ordered subset expectation-maximization algorithm with the following settings: four iterations, six subsets, full detector model, low regularization, spike filter on, and a voxel size of 0.6 mm. Decay, scatter, and attenuation corrections were applied during the reconstruction. The final reconstructed SPECT and CT images had matrix sizes of 142 × 142 × 163 mm³ and voxel sizes of 0.6 × 0.6 × 0.6 mm³ for further analysis. The PMOD software version 3.8 (PMOD Technologies, Zurich, Switzerland) was used to process the SPECT and CT images, including activity normalization and registration.

### Image analysis

Volumes of interest (VOIs) were drawn manually on SPECT- and CT-fused images of the major organs (stomach, intestine, liver, heart, kidney, and urinary bladder). Care was taken to ensure that VOIs did not overlap. The VOIs were applied to the corresponding organs on the SPECT images to estimate the radioactivity of each organ. The measured activity (kBq/cc) was normalized to the total injected activity to calculate the percentage of the injected dose per Gram (%ID/g). The biodistribution data were plotted over time to generate time-activity curves (TACs). Data were expressed as mean ± standard error of the mean (SEM). The pharmacokinetic parameters of radioiodine in each organ, including peak concentration (C_max_), time to reach C_max_ (T_max_), half-life (T_1/2_), and area under the curve (AUC) were quantitatively evaluated using the TACs of the organs of interest.

### Statistical analysis

All statistical analyses were performed using Prism software (GraphPad Software version 5.0, La Jolla, CA, USA). Data are expressed as mean values ±standard error of the mean, and comparisons of quantitative data between the two groups were analyzed using an unpaired *t*-test. Statistical significance was set at *p* < 0.05.

## Results

### Preparation of [^123^I]I-paclitaxel, [^123^I]APNs and L-[^123^I]APNs

Radioiodination of paclitaxel ([^123^I]I-paclitaxel) was successfully achieved through a conventional electrophilic aromatic substitution reaction on activated paclitaxel using a *tert*-butylstannyl moiety and the standard chloramine-T oxidation method. The radiosynthesis process took approximately 80 min, and the radioactivity yield after HPLC separation was found to be 29.8% ± 2.6% (Figure 2A, n = 14, non-decay corrected). The radiochemical purity was greater than 99% (Figure 2B; *t*_*R*_ = 8.4 min). No significant radiolysis of [^123^I]I-paclitaxel was observed in absolute ethanol after 4 h at room temperature. Subsequently, an ethanol solution containing [^123^I]I-paclitaxel was mixed with an albumin solution in a 1:10 (v/v) ratio, inducing the self-assembly of albumin and forming [^123^I]APNs. The desired product was obtained using a desolvation technique, yielding 33.2% ± 4.7% (n = 11, non-decay corrected) of radioactivity yield calculated from the used [^123^I]I-paclitaxel radioactivity. The preparation time for the [^123^I]iodo-paclitaxel-loaded albumin was approximately 180 min. No significant differences were observed in the separation methods between ultrafiltration and the use of the PD-10 column (31.2% ± 6.1% vs. 33.2% ± 4.7%, n > 3, non-decay corrected).

**FIGURE 3:**
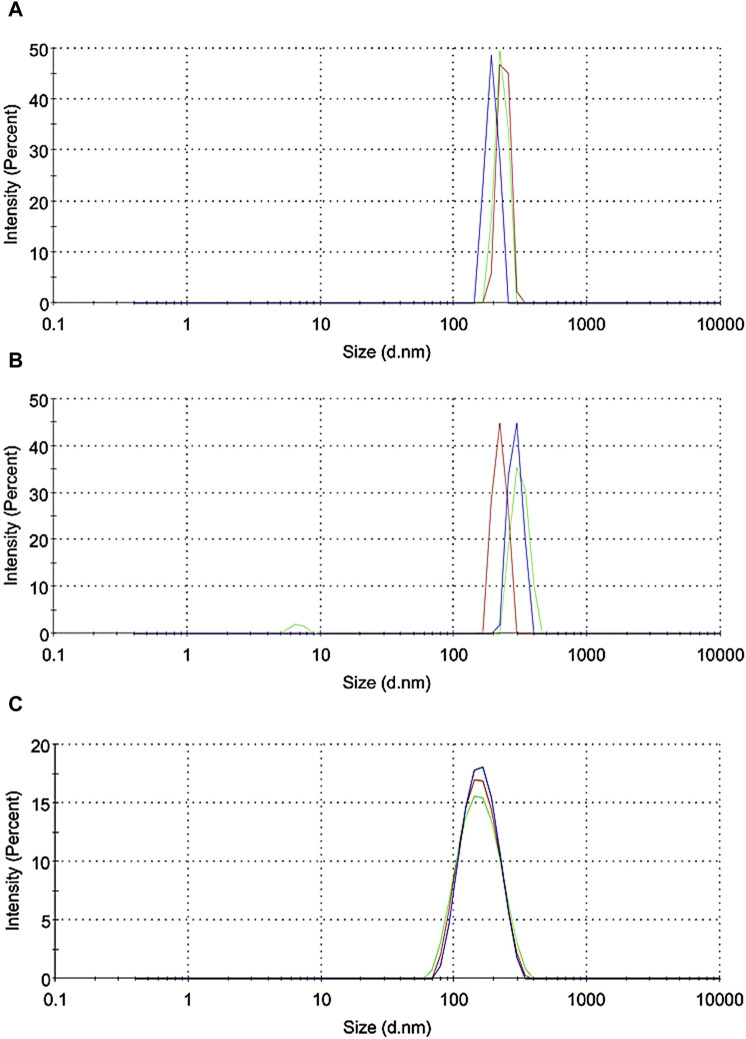
Dynamic light scattering data (*n* = 3) of [^123^I]APNs **(A)**, L-[^123^I]APNs **(B)**, and Abraxane **(C)**. [^123^I]APNs, albumin-[^123^I]iodo-paclitaxel nanoparticles; L-[^123^I]APNs, liposome-encapsulated albumin-[^123^I]iodo-paclitaxel nanoparticles.

Subsequently, the [^123^I]APNs formed were encapsulated in liposomes by hydrating the dried lipid thin film to produce L-[^123^I]APNs. Results of dynamic light scattering (DLS) measured in phosphate buffered saline showed 193 ± 20 nm for [^123^I]APNs (Figure 3A) and 241 ± 24 nm for L-[^123^I]APNs (Figure 3B). The size of L-[^123^I]APNs was slightly larger than that of [^123^I]APNs or Abraxane (163 ± 47 nm, Figure 3C), suggesting that the L-[^123^I]APNs was loaded within the vesicular structure. The encapsulation efficiency (EE) of L-[^123^I]APNs was 82.8% ± 7.8%.

### *In vivo* tissue pharmacokinetics

**FIGURE 4:**
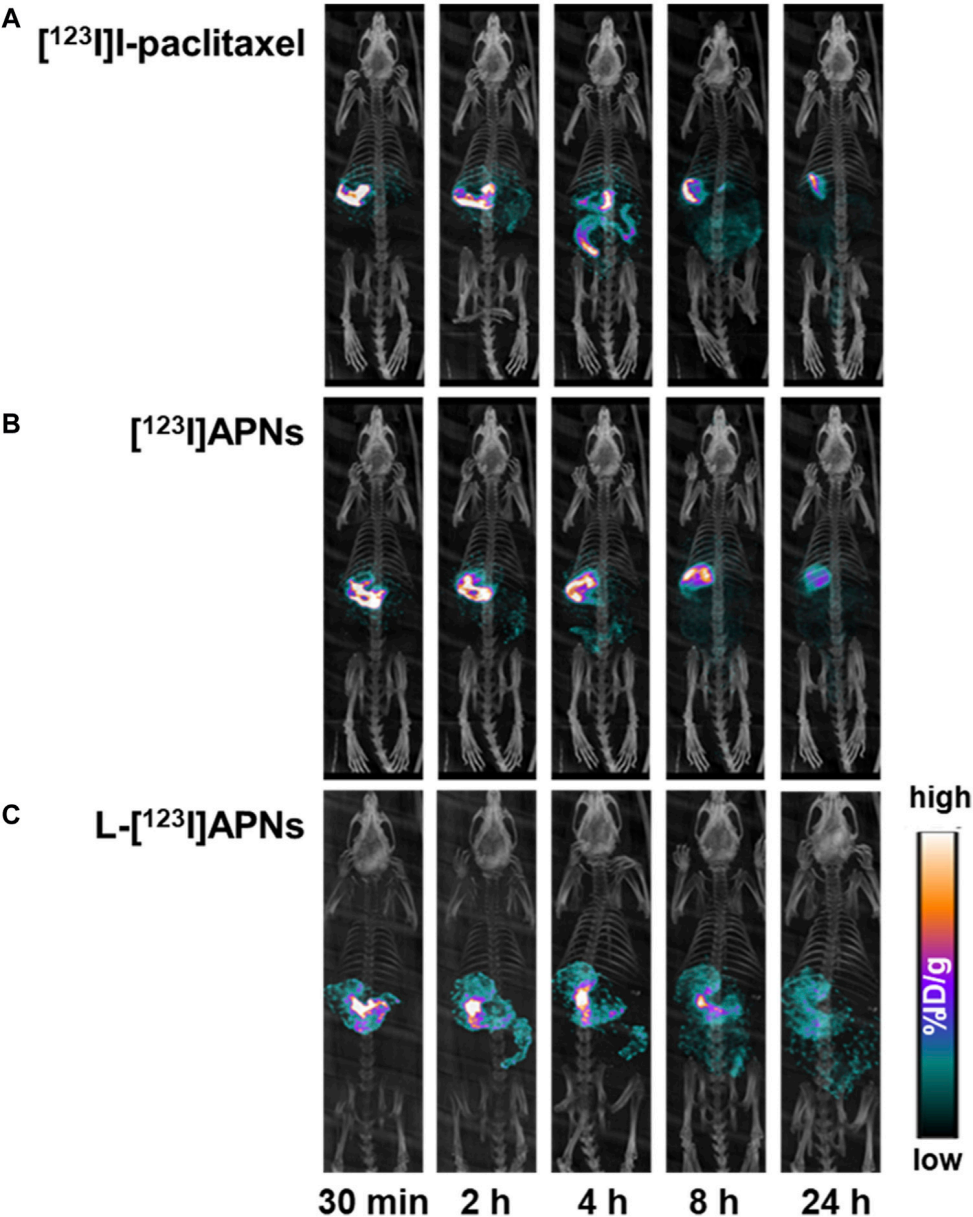
Representative single photon emission computed tomography/computed tomography images over time of [^123^I]I-paclitaxel **(A)**, [^123^I]APNs **(B)**, and L-[^123^I]APNs **(C)**. [^123^I]APNs, albumin-[^123^I]iodo-paclitaxel nanoparticles; L-[^123^I]APNs, liposome-encapsulated albumin-[^123^I]iodo-paclitaxel nanoparticles.

**FIGURE 5:**
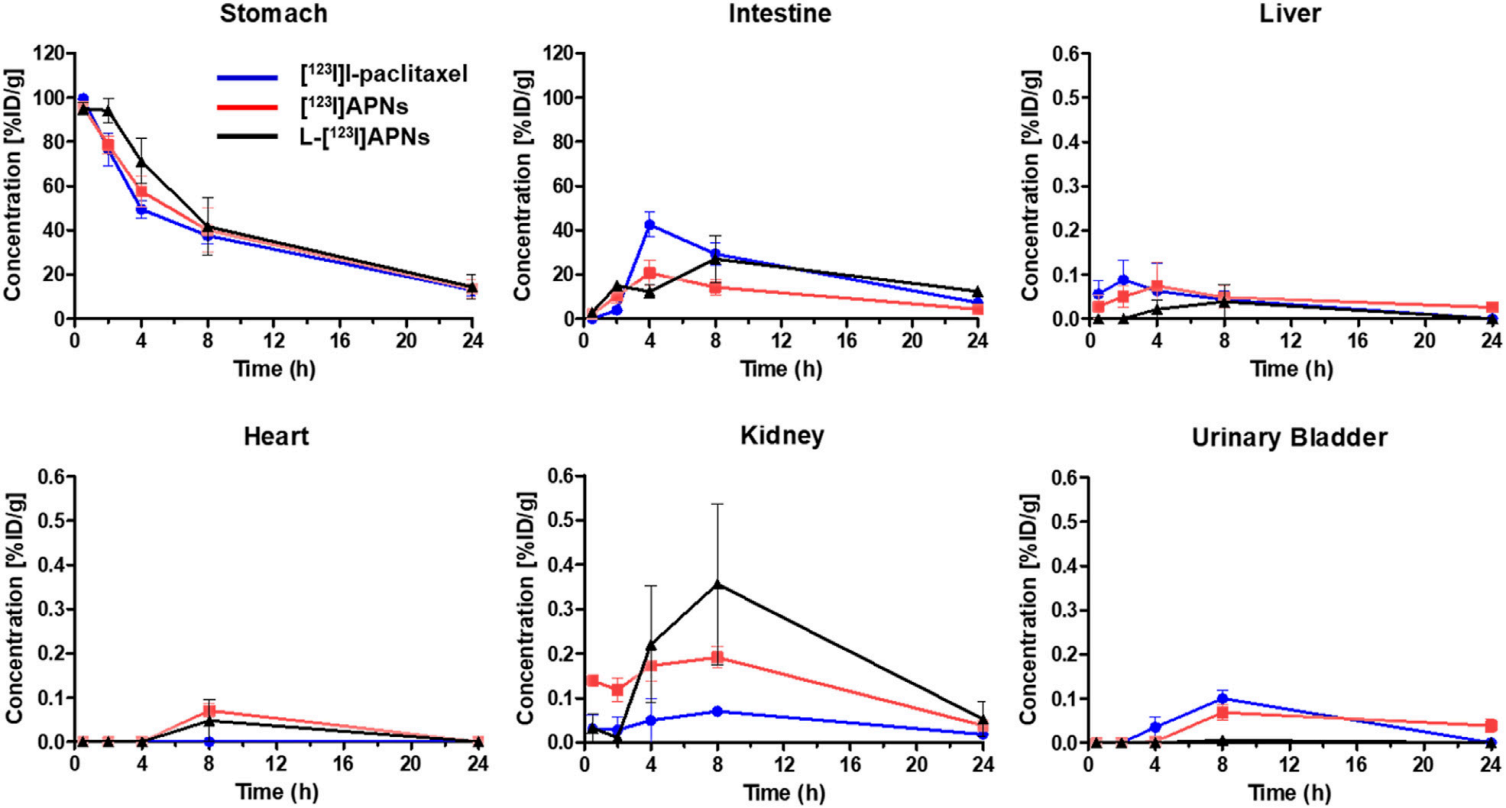
Time-activity curves of [^123^I]I-paclitaxel (blue), [^123^I]APNs (red), and L-[^123^I]APNs (black) calculated from whole-body single photon emission computed tomography/computed tomography images. Please note the differences in scaling, with liver, hearth, kidney, and urinary bladder having uptake <1 %ID/g. [^123^I]APNs, albumin-[^123^I]iodo-paclitaxel nanoparticles; L-[^123^I]APNs, liposome-encapsulated albumin-[^123^I]iodo-paclitaxel nanoparticles.

To evaluate the *in vivo* tissue distribution of [^123^I]I-paclitaxel, [^123^I]APNs, and L-[^123^I]APNs in normal rats, radioisotope-labeled drugs were administered via the gastric subserosal route. Whole-body SPECT/CT scans were acquired using a small animal-dedicated SPECT/CT system at 30 min and 2, 4, 8, and 24 h after each injection of [^123^I]I-paclitaxel, [^123^I]APNs, or L-[^123^I]APNs. The acquired SPECT images were reconstructed using the iterative three-dimensional ordered subset expectation-maximization algorithm into three-dimensional images by combining them with the CT images. Representative SPECT/CT images as time and time–radioactivity profiles generated for each interest organ determined from whole-body SPECT images are shown in Figures 4, 5, respectively. In addition, pharmacokinetic parameters such as T_1/2_, T_max_, AUC, and C_max_ were analyzed based on the quantified biodistribution data of each organ over time (Table 1), and the excretion routes were also estimated (Moon et al., 2021; Park et al., 2022; Park et al., 2023).

**TABLE 1:** Pharmacokinetic parameters of [^123^I]I-paclitaxel, [^123^I]APNs and L-[^123^I]APNs after gastric subserosal administration in healthy rats^a^.

	Organ	T_1/2_ [h]	C_max_ [%ID/g]	T_max_ [h]	AUC_0-t_ [%ID/g x h]
[^123^I]I-paclitaxel	Stomach	3.23 ± 0.34	99.5 ± 0.3	0.50 ± 0.00	838 ± 44
	Intestine	5.26 ± 2.90	44.3 ± 3.9	5.33 ± 1.33	487 ± 44
	Liver	2.14 ± 0.16	0.12 ± 0.07	1.67 ± 1.17	0.93 ± 0.54
	Heart	-	-	-	-
	Kidney	2.13 ± 0.90	0.03 ± 0.03	0.50 ± 0.00	1.07 ± 0.14
	Urinary Bladder	1.97 ± 0.00	0.04 ± 0.04	3.00 ± 2.50	1.11 ± 0.39
[^123^I]APNs	Stomach	5.01 ± 1.06	95.7 ± 0.9	0.50 ± 0.00	893 ± 160
	Intestine	7.27 ± 1.03	20.8 ± 5.7	4.00 ± 0.00	261 ± 60
	Liver	2.15 ± 0.52	0.11 ± 0.07	2.67 ± 0.67	1.24 ± 0.50
	Heart	1.97 ± 0.00	0.04 ± 0.04	3.00 ± 2.50	0.77 ± 0.21
	Kidney	-	0.20 ± 0.03	6.67 ± 1.33	3.05 ± 0.36
	Urinary Bladder	2.32 ± 0.35	0.02 ± 0.02	8.33 ± 7.83	1.11 ± 0.26
L-[^123^I]APNs	Stomach	6.11 ± 3.21	99.0 ± 1.4	1.50 ± 0.50	983 ± 167
	Intestine	-	30.7 ± 7.3	6.67 ± 1.33	436 ± 95
	Liver	-	0.06 ± 0.06	3.00 ± 2.50	0.66 ± 0.66
	Heart	-	0.05 ± 0.05	3.00 ± 2.50	0.68 ± 0.68
	Kidney	-	0.23 ± 0.14	4.17 ± 2.17	5.35 ± 2.47
	Urinary Bladder	-	0.01 ± 0.01	3.00 ± 2.50	0.06 ± 0.06

^a^ Data shown are mean values ± Standard Error of the Mean (*n* = 3).

Paclitaxel, APNs, and L-APNs showed a high cumulative distribution in the stomach. In decreasing order, the AUCs were 983 ± 167 for L-APNs, 893 ± 160 for APNs, and 838 ± 44 for paclitaxel. Although the differences were not statistically significant, likely due to the limited number of animals in each study group, (*p* = 0.4, L-APNs vs. APNs; *p* = 0.18, L-APNs vs. Paclitaxel), the combination for L-APNs of highest AUC and initially flat curves in all organs, seems to indicate a prolonged and sustained presence of L-APNs in the stomach. Drugs administered through the gastric subserosal route were mostly distributed in the stomach and intestines, with a low intake level (<1%) in other major organs. Paclitaxel, APNs, and L-APNs were mainly excreted in the feces through the intestines, and the time to reach the maximum concentration in the intestine for L-APNs was the longest at 6.67 h, followed by paclitaxel at 5.33 h and APNs at 4.00 h. These findings underscore the potential of L-APNs for targeted and prolonged drug delivery to the stomach, possibly reducing systemic exposure and side effects. L-APNs (C_max_ = 0.23 ± 0.14 %ID/g) showed relatively high uptake in the kidneys compared to other drugs, followed by APNs (0.20 ± 0.03 %ID/g) and paclitaxel (0.03 ± 0.03 %ID/g) in that order.

On the other hand, the uptake of free I-123 in the thyroid region, which can predict *in vivo* instability, was not observed for any of the drugs, [^123^I]I-paclitaxel, [^123^I]APNs, and L-[^123^I]APNs. The *in vivo* stability seemed to be very high, allowing for unimpeded evaluation of their distribution *in vivo*.

## Discussion

In this study, we successfully synthesized [^123^I]I-paclitaxel and prepared L-[^123^I]APNs containing [^123^I]APNs. The resulting material exhibited high stability, confirming its potential for *in vivo* applications. The somewhat longer preparation time for [^123^I]APNs from radioiodination, approximately 260 min, was acceptable in terms of the half-life of iodine-123 (*t*_*1/2*_ = 13 h) and in the context of the objectives of our study.

Akamo et al. compared the effectiveness of delivering adriamycin (ADR) to perigastric lymph nodes using gastric submucosal injection of liposomal adriamycin (Lipo-ADR) and intravenous administration of free ADR (F-ADR) (Akamo et al., 1994). The study involved 34 gastric carcinoma patients who received Lipo-ADR via endoscopic injection into the gastric submucosa adjacent to the primary tumor and 18 patients who received F-ADR intravenously. The results revealed that following Lipo-ADR injection, the concentration of ADR in the primary and secondary drainage lymph nodes was significantly higher than in other regional lymph nodes.

Liposomes are lipid-based structures that encapsulate drugs within lipid membranes. In the evaluation of the *in vivo* tissue pharmacokinetics, our findings indicated a notable accumulation of APNs and L-APNs in the stomach. This observation aligns with the subserosal gastric injection route, which was selected because it efficiently targets gastric tissues. The area under the curve (AUC) associated with L-APNs is noteworthy, suggesting their potential for extended release and sustained therapeutic effects. This extended release was further supported by the delay in reaching the maximum concentration in the intestines, with L-APNs requiring the longest duration.

Okamoto et al. employed liposomes to address the challenge of the low water solubility of paclitaxel (Okamoto et al., 2019). In this study, we explored the *in vivo* antitumor effects of APNs in a mouse model of subcutaneously inoculated pancreatic cancer cells. These results demonstrate that APNs effectively accumulated at the tumor site, presumably through an enhanced permeability retention effect, and exhibited significant antitumor activity.

In the present study, the low distribution of these drugs in other major organs of the body (<1%) suggests that the subserosal injection route effectively minimizes systemic distribution, emphasizing the potential for localized therapy with reduced systemic side effects. These results highlight the potential of our approach for delivering paclitaxel and other lipophilic anticancer drugs, offering a strategy to enhance their therapeutic impact while minimizing systemic exposure. Thus, L-APNs have the potential to serve as a novel method for preparing paclitaxel for gastric cancer treatment. The application of nuclear medicine and molecular imaging technology to evaluate the distribution and pharmacokinetics of these agents *in vivo* demonstrated the translational potential of this research in the field of cancer treatment.

It is important to note that further studies, including toxicity assessments, efficacy evaluations, and clinical trials, will be necessary to translate these findings into clinical practice. Nonetheless, the promising results obtained in this study provide a strong foundation for future investigations into developing novel localized anticancer therapies.

The current study had some limitations. Paclitaxel and [^123^I]iodo-paclitaxel ([^123^I]I-paclitaxel) are structurally slightly different. [^18^F]Fluoro-paclitaxel, which has relatively smaller structural change, has a very similar distribution to paclitaxel (Kiesewetter et al., 2003). However, [^18^F]fluoro-paclitaxel was unsuitable for this study because it has a relatively short half-life of 110 min. There may be differences in body distribution between paclitaxel and [^123^I]iodo-paclitaxel, but these could be considered minor differences in the present study using identical conditions. Additionally, when [^123^I]I-paclitaxel is broken down *in vivo*, free iodide is absorbed into thyroid tissues. Considering that all three radiotracers, [^123^I]I-paclitaxel, [^123^I]APNs, and L-[^123^I]APNs, were barely absorbed, the *in vivo* stability appeared to be very high; thus, there were no problems in evaluating their distribution *in vivo*. Another limitation is that the sample size used in this preliminary experiment was relatively small, which may have limited the accuracy of the distribution of these substances in the body. Nevertheless, this study, which assessed tissue pharmacokinetics using image-based nuclear medicine and molecular imaging techniques, indicated the potential of L-APNs.

## Conclusion

These initial findings imply the potential of L-APNs as a novel preparation method of paclitaxel for regional treatment of gastric cancer. These results indicate that L-APNs exhibit favorable distribution and accumulation in the stomach, making them a potentially valuable approach for localized treatment. Further preclinical and clinical studies are warranted to explore the full potential and efficacy of L-APNs in gastric cancer therapy.

## Data Availability

The raw data supporting the conclusion of this article will be made available by the authors, without undue reservation.
